# Integration of Nuclear, Clinical, and Genetic Features for Lung Cancer Subtype Classification and Survival Prediction Based on Machine- and Deep-Learning Models

**DOI:** 10.3390/diagnostics15070872

**Published:** 2025-03-28

**Authors:** Bin Xie, Mingda Mo, Haidong Cui, Yijie Dong, Hongping Yin, Zhe Lu

**Affiliations:** 1 School of Information Science and Technology, Hangzhou Normal University, Hangzhou 311121, China; xiebin@hznu.edu.cn (B.X.); 2022112011059@stu.hznu.edu.cn (M.M.); 2 Department of Breast Surgery, The First Affiliated Hospital, School of Medicine, Zhejiang University, Hangzhou 311121, China; cuihaidong79@163.com; 3 School of Software Technology, Zhejiang University, Ningbo 315048, China; r_raise_semitone@zju.edu.cn; 4 School of Basic Medical Sciences, Hangzhou Normal University, Hangzhou 311121, China; 20021528@hznu.edu.cn; 5 Zhejiang Key Laboratory of Medical Epigenetics, Hangzhou Normal University, Hangzhou 311121, China

**Keywords:** machine learning, deep learning, lung cancer, subtype classification, overall survival prediction

## Abstract

**Objectives:** Lung cancer is one of the most prevalent cancers worldwide. Accurately determining lung cancer subtypes and identifying high-risk patients are helpful for individualized treatment and follow-up. Our study aimed to establish an effective model for subtype classification and overall survival (OS) prediction in patients with lung cancer. **Methods:** Histopathological images, clinical data, and genetic information of lung adenocarcinoma and lung squamous cell carcinoma cases were downloaded from The Cancer Genome Atlas. An influencing factor system was optimized based on the nuclear, clinical, and genetic features. Four machine-learning models—light gradient boosting machine (LightGBM), extreme gradient boosting (XGBoost), random forest (RF), and adaptive boosting (AdaBoost)—and three deep-learning models—multilayer perceptron (MLP), TabNet, and convolutional neural network (CNN)—were employed for subtype classification and OS prediction. The performance of the models was comprehensively evaluated. **Results:** XGBoost exhibited the highest area under the curve (AUC) value of 0.9821 in subtype classification, whereas RF exhibited the highest AUC values of 0.9134, 0.8706, and 0.8765 in predicting OS at 1, 2, and 3 years, respectively. **Conclusions:** Our study was the first to incorporate the characteristics of nuclei and the genetic information of patients to predict the subtypes and OS of patients with lung cancer. The combination of different factors and the usage of artificial intelligence methods achieved a small breakthrough in the results of previous studies regarding AUC values.

## 1. Introduction

According to the global cancer statistics from the International Agency for Research on Cancer, lung cancer is one of the most prevalent cancers worldwide. There were approximately 2.5 million new lung cancer cases in 2022, accounting for 12.4% of all new cancer cases. The number of lung cancer deaths in 2022 was approximately 1.8 million, representing 18.7% of total cancer fatalities and establishing it as a leading cause of cancer-related death [1]. Lung cancer primarily consists of two types: non-small cell lung cancer (NSCLC) and small cell lung cancer. NSCLC represents more than 85% of all lung cancer cases, with its primary histological subtypes being lung adenocarcinoma (LUAD) and lung squamous cell carcinoma (LUSC). Seventy percent of lung cancer cases are diagnosed at advanced stages, and treatment options are often limited [2]. Therefore, accurately determining lung cancer subtypes and identifying high-risk patients can facilitate individualized treatment and follow-up. Histopathological examination is the gold standard for identifying the subtypes of lung cancer, but it relies on the experience of pathologists and is time-consuming [3,4]. Moreover, in predicting the survival of patients with lung cancer, common linear methods such as nomograms may fail to capture intricate nonlinear relationships in high-dimensional datasets [5,6].

A growing number of studies have attempted to apply machine- and deep-learning models for subtype classification and survival prediction in patients with NSCLC [7]. For subtype classification, for example, Han et al. collected positron emission tomography (PET)/computed tomography (CT) images of 867 and 552 patients with LUAD and LUSC, respectively, from Peking University Cancer Hospital. They used VGG16 for classification based on first-order intensity statistics and texture features, achieving an accuracy of 84.1% and an area under the curve (AUC) of 0.903 [8]. Kriegsmann et al. collected image data of 499 LUAD and 440 LUSC cases from Heidelberg University Thoracic Hospital. Utilizing imaging features and a random forest (RF) model for classification, they obtained an accuracy of 90.6% [9]. However, several studies showed limitations related to the use of test methods. Some studies split different images from the same patient into training and testing sets—the use of similar data for training and subsequent testing may have resulted in high accuracy [10]; in other studies, the images used for both training and testing datasets were derived from a common set of tissue slices, from which more images were expanded using different post-processing methods [11]. For example, in one study, the dataset was expanded by using techniques such as rotation and flipping before splitting them into training, validation, and testing sets [12]. Furthermore, some studies utilized datasets with limited sample sizes—under 350 cases of patients [13,14,15,16,17]. Some studies had adequate samples; however, model performance was compromised with AUC values under 0.9, limiting the practical application of the model. For example, Hyun et al. collected data from 210 LUAD and 186 LUSC cases from the Samsung Medical Center in Korea and used a logistic regression model to classify subtypes based on PET/CT radiomics and clinical features, including age, sex, tumor size, and smoking status, achieving an accuracy of 76.9% and an AUC of 0.859 [18]. Song et al. downloaded the CT images of 700 lung cancer cases (including 496 LUAD and 204 LUSC cases) from The Cancer Imaging Archive (TCIA) platform and used the bagging–Adaptive Boosting (AdaBoost)–support vector machine approach for subtype classification based on CT intensity, texture, and filtered image features, achieving an AUC of 0.823 [19].

For overall survival (OS) prediction, some studies have also shown issues with small datasets [20,21,22,23]. For example, He et al. downloaded only 186 CT images of NSCLC cases from the TCIA platform and predicted survival status using intensity, shape, texture, and wavelet in combination with an RF model, achieving an AUC of 0.9296 [20]. Chaddad et al. downloaded the data of 315 NSCLC cases from the TCIA platform, and they used CT image features, demographic data, and tumor, node, metastasis (TNM) staging variables to predict whether the patients’ survival would be above, equal to, or below the median survival time with an RF model, achieving an AUC of 0.7617 [22]. In contrast, while some studies employed a sufficient number of samples, their performance remained compromised, with AUC values under 0.75 or even under 0.7 [24,25,26]. Thus, a more effective model is needed.

Changes in the distribution, appearance, size, morphology, and arrangement of the nuclei of cancer cells have been shown to predict cancer aggressiveness. Petersen et al. showed that different lung cancer types had different nuclear sizes. In NSCLC, especially LUAD, the size of nuclei correlated significantly with the grading and survival of patients [27]. In addition, Sigel et al. suggested that several cytomorphologic features, such as nuclear size, chromatin pattern, and nuclear contours, could be used in a scoring system as they correlated well with histologic grade and prognosis [28]. However, their study only showed the correlation without proposing a model. Based on the attributes of individual nuclei (e.g., shape, size, and texture), Lu et al. classified the long-term versus short-term survival of patients with early-stage NSCLC, yielding a mean AUC of 0.68 in the training cohort [29]. The utilization of nuclear features to predict the OS of patients with lung cancer remains limited.

To establish a suitable model with optimal performance in lung cancer subtype classification and OS prediction, we constructed a large dataset consisting of 1252 LUAD and LUSC cases, integrating nuclear, clinical, and genetic features for a total of 95 variables. Uncorrelated factors were excluded using Pearson’s correlation coefficient (PCC) analysis. To analyze the performance of different models, we employed four machine-learning models (light gradient boosting machine [LightGBM], extreme gradient boosting [XGBoost], RF, and AdaBoost) and three deep-learning models (multilayer perceptron [MLP], TabNet, and convolutional neural network [CNN]) for subtype classification as well as prediction of survival at 1, 2, and 3 years among patients with LUAD and LUSC. In addition, we assessed the performances of these models using metrics such as precision, accuracy, AUC, recall, F1-score, and learning duration. Through this multidimensional assessment, we aimed to identify an optimal model for subtype classification and OS prediction in patients with NSCLC.

## 2. Data and Methods

### 2.1. Data Source

The TCGA is a program initiated by the National Cancer Institute and the National Human Genome Research Institute of the United States in 2006. It contains extensive clinical and genetic information as well as histopathological images of 33 types of cancer and over 10,000 cancer cases [30].

To study LUAD and LUSC, we downloaded the corresponding manifest files from the TCGA website (https://portal.gdc.cancer.gov/) accessed on 10 October 2022 and used GDC-Client software (Version 1.6.1) to download the histopathological images for LUADs and LUSCs, along with their related clinical features and copy number variation (CNV) and single nucleotide variant (SNV) information. The total number of downloaded images was 5548 from 1252 patients, including 525 patients with LUAD and 727 patients with LUSC.

### 2.2. Overview of the Analysis Scheme

**Figure 1:**
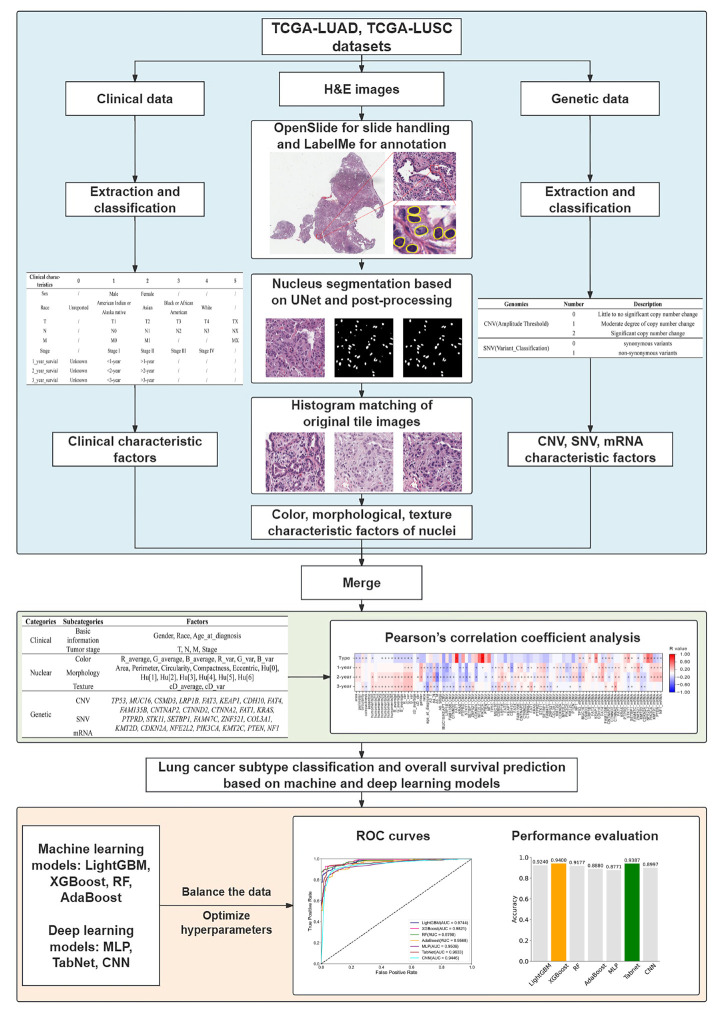
An overview of the analysis scheme. *: features with |r| > 0.05 and *p* < 0.05; +: high correlation features with |r| > 0.8 in the heatmap of Person’s correlation coefficient analysis.

The workflow of the analysis is shown in Figure 1. First, we downloaded the clinical data, Hematoxylin and eosin (H&E) staining images, and genetic features from the TCGA datasets for LUAD and LUSC (TCGA-LUAD, TCGA-LUSC). Second, a comprehensive feature dataset was obtained by merging the data, and features with low correlations were eliminated using PCC analysis. Finally, using four machine-learning and three deep-learning models, the classification of lung cancer subtypes and survival prediction were conducted based on balanced training samples and automated hyperparameter optimization, followed by an evaluation of the performance of the models.

### 2.3. UNet-Based Automated Segmentation of Lung Cancer Cell Nuclei

#### 2.3.1. OpenSlide for Slide Handling and LabelMe for Annotation

Training machine- and deep-learning models directly on whole-slide images (WSIs) with gigapixel resolution presents significant computational challenges given the current computational resources. To address this issue, we employed OpenSlide’s leveling and tiling techniques. OpenSlide (Version 1.3.1) [31] (https://openslide.org/) is an open-source C library used for reading and processing WSIs in digital pathology and is a crucial tool for handling and analyzing large-scale pathology image data. Using the Python interface provided by OpenSlide, we segmented the lung cancer WSIs into smaller tile images, each with a uniform size of 1024 × 1024 pixels.

The LabelMe software (Version 5.3.1) [32] was used for annotation to obtain training samples of lung cancer cells. LabelMe, an open-source tool for online image annotation (https://github.com/labelmeai/labelme accessed on 19 March 2025), was created by the Computer Science and Artificial Intelligence Laboratory at the Massachusetts Institute of Technology. It is widely used to manually annotate objects and regions in images, thereby facilitating the setup of datasets for various computer vision tasks. Based on the segmented images, we marked the locations and shapes of the nuclei of lung cancer cells to generate their corresponding mask images.

#### 2.3.2. UNet-Based Nucleus Segmentation

The UNet model [33] has demonstrated outstanding performance in various biomedical segmentation tasks and has achieved excellent results across different applications. In our study, the UNet model was built using Anaconda 2023.9, Python 3.9.18, and Torch 1.8.0+cu111 within the PyCharm 2023.3 integrated development environment (https://github.com/xiaopeng-liao/Pytorch-UNet accessed on 19 March 2025).

We input lung cancer cell nucleus mask images annotated with LabelMe software into the UNet model for training and then used the trained model to perform automated segmentation of lung cancer cell nuclei in unlabeled lung cancer images.

#### 2.3.3. Post-Processing of Segmentation Results Using Otsu’s Thresholding Method

Compactness measures the similarity of a shape to a circle of the same area.

(1) $$C=\frac{P^{2}}{A}$$

It is defined as the ratio of the square of the perimeter (*P*) to the area (*A*) of the shape, where C represents compactness. Higher compactness indicates a more irregular shape, with a circle having the minimum compactness.

Otsu’s thresholding method [34] is a nonparametric, unsupervised automatic threshold-selection technique used for image segmentation that reliably selects the optimal threshold based on a discriminative criterion. Specifically, for each image, we calculated the area and compactness of the nuclei segmented by UNet and dynamically generated the corresponding area and compactness thresholds using Otsu’s method. Otsu’s method determines thresholds adaptively based on the features in each image; thus, the area and compactness thresholds are not fixed for all the images. To obtain these thresholds, we first computed the histogram distribution of the nucleus area and compactness values within each image, and then Otsu’s method was applied to these histograms to determine the optimal area and compactness thresholds that minimized intra-class variance. Based on the obtained thresholds, nuclei with an area smaller than the area threshold and compactness greater than the compactness threshold were removed, whereas those with good prediction performance were retained. This process resulted in the generation of a mask image corresponding to the nuclei of lung cancer cells.

### 2.4. Construction of Clinical, Nuclear, and Genetic Feature Systems

We integrated clinical information, findings from histopathological images, and genetic information such as CNV, SNV, and messenger RNA (mRNA) data to construct a multidimensional-based evaluation system. We picked the 20 most frequently mutated genes in both LUAD and LUSC and then filtered and obtained the CNV, SNV, and mRNA data of the 27 common genes between LUAD and LUSC, which were as follows: TP53, MUC16, CSMD3, LRP1B, FAT3, KEAP1, CDH10, FAT4, FAM135B, CNTNAP2, CTNND2, CTNNA2, FAT1, KRAS, PTPRD, STK11, SETBP1, FAM47C, ZNF521, COL3A1, KMT2D, CDKN2A, NFE2L2, PIK3CA, KMT2C, PTEN, and NF1.

#### 2.4.1. Clinical Features

The clinical information extracted from the TCGA included data for sex, race, age at diagnosis, tumor stage (TNM classification and stage), OS, and current survival status (CSS). Next, we determined the survival status of each patient at 1, 2, and 3 years based on the OS and CSS and assigned them numerical values, with 0 indicating unknown, 1 indicating death, and 2 indicating survival. All string values of clinical information were converted to numerical values (e.g., 0, 1, 2), as listed in Table 1, to facilitate their use as inputs for machine- and deep-learning models.

#### 2.4.2. Nuclear Features

##### Color Parameters

Histopathological images typically show color variations resulting from differences in the staining process and the use of different microscopic equipment. Histogram matching [35] is an effective method for color normalization that aligns the color characteristics of one image with those of another. However, careful consideration is required when selecting the reference image because choosing an unsuitable image may affect the normalization results [36].

**Figure 2:**
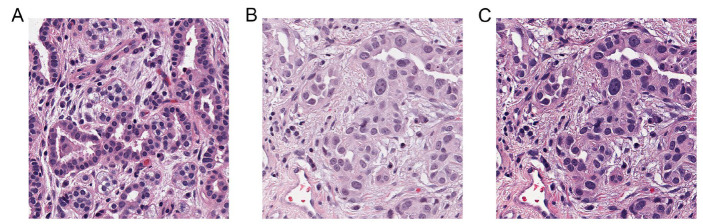
Histogram-matching process. (**A**) The reference image selected by the expert for histogram matching, (**B**) an image with color discrepancies before histogram matching, and (**C**) the image after histogram matching. Magnification: ×400.

In our study, a pathologist selected the image with optimal staining as the reference image (Figure 2A). All tile images were processed using histogram matching to eliminate color discrepancies (Figure 2B,C). Subsequently, we obtained the color parameters for each nucleus, including the mean and variance of the RGB values within the nucleus.

##### Morphological Parameters

Morphological parameters (area, perimeter, circularity, compactness, eccentricity, and Hu moments) are key metrics used to describe the shape and characteristics of cell nuclei.

Circularity quantifies the resemblance between a shape and a circle. It is defined as the ratio of the area to the square of the perimeter, where *C* represents circularity, *A* is the area of the shape, and *P* is the perimeter of the shape:

(2) $$C=\frac{4\pi A}{P^{2}}$$

Eccentricity is an important measure for describing the characteristics of an ellipse, and it represents the ratio of the distance from the center to the focus of the ellipse to the length of the semi-major axis. Here, *E* denotes eccentricity, with *a* representing the length of the semi-major axis and *b* indicating the length of the semi-minor axis:

(3) $$E=\frac{\sqrt{a^{2}-b^{2}}}{a}$$

Owing to their translation, rotation, scale, and reflection invariances, Hu moments are often applied in tasks such as image matching, target detection, and classification. Hu moments are a combination of seven numbers, each derived from a central moment. First, the geometric and central moments of the image are calculated as follows:

The geometric moment of a 2D image *f*(*x*,*y*) is defined as $m_{pq}$, where *p* and *q* are non-negative integers:

(4) $$m_{pq}=\sum_{x}\sum_{y}x^{p}y^{q}\int \left(x,y\right)$$

The central moment of the image is defined as $\mu_{pq}$, where $\bar{x}=\frac{m_{10}}{m_{00}}$ and $\bar{y}=\frac{m_{01}}{m_{00}}$ are the coordinates of the image centroid:

(5) $$\mu_{pq}=\sum_{x}\sum_{y}{\left(x-\bar{x}\right)}^{p}{\left(y-\bar{y}\right)}^{q}\int \left(x,y\right)$$

The central moment is normalized and defined as $\eta_{pq}$, where $\gamma =\frac{p+q}{2}+1$:

(6) $$\eta_{pq}=\frac{\mu_{pq}}{\mu_{00}^{\gamma }}$$

Hu moments are invariant moments calculated on the basis of the normalized central moments and are expressed as follows:

(7) $${Hu}_{0}=\eta_{20}+\eta_{02}$$

(8) $${Hu}_{1}={{(\eta }_{20}-\eta_{02})}^{2}+4\eta_{11}^{2}$$

(9) $${Hu}_{2}={{(\eta }_{30}-{3\eta }_{12})}^{2}+{{(3\eta }_{21}-\eta_{03})}^{2}$$

(10) $${Hu}_{3}={{(\eta }_{30}+\eta_{12})}^{2}+{{(\eta }_{21}+\eta_{03})}^{2}$$

(11) $${Hu}_{4}={(\eta }_{30}-{3\eta }_{12}){(\eta }_{30}+\eta_{12})\left[{{(\eta }_{30}-{3\eta }_{12})}^{2}-3{{(\eta }_{21}+\eta_{03})}^{2}\right]\;+{(3\eta }_{21}-\eta_{03}){(\eta }_{21}+\eta_{03})\left[3{{(\eta }_{30}+\eta_{12})}^{2}-{{(\eta }_{21}+\eta_{03})}^{2}\right]$$

(12) $${Hu}_{5}={(\eta }_{20}-\eta_{02})\left[{{(\eta }_{30}+\eta_{12})}^{2}-{{(\eta }_{21}+\eta_{03})}^{2}\right]\;+{{4\eta }_{11}(\eta }_{30}+\eta_{12}){(\eta }_{21}+\eta_{03})$$

(13) $${Hu}_{6}={(3\eta }_{21}-\eta_{03}){(\eta }_{21}+\eta_{03})\left[3{{(\eta }_{30}+\eta_{12})}^{2}-{{(\eta }_{21}+\eta_{03})}^{2}\right]\;-{(\eta }_{30}-{3\eta }_{12}){(\eta }_{21}+\eta_{03})\left[3{{(\eta }_{30}+\eta_{12})}^{2}-{{(\eta }_{21}+\eta_{03})}^{2}\right]$$

##### Texture Parameters

Texture parameters are commonly used in image analysis to capture details and complex structural information in images. The 2D Discrete Wavelet Transform decomposes an image signal into four components: an approximation component (cA) and three detail components (cH, cV, and cD). The diagonal detail component (cD) contains the high-frequency components of the image and primarily captures the edges and detailed information along the diagonal direction.

Assuming the original image as *I*, the wavelet filters *g* for low-pass filters and *h* for high-pass filters were used for the computation. A 1D wavelet transform was applied to each row I(i,:), resulting in a horizontal low-frequency component $A_{h}$ and a horizontal high-frequency component $D_{h}$. Here, *i* is the row index, *j* is the column index, and *k* is the summation index:

(14) $$A_{h}\left(i,j\right)=\sum_{k}I\left(i,k\right)*g\left[2j-k\right]$$

(15) $$D_{h}\left(i,j\right)=\sum_{k}I\left(i,k\right)*h\left[2j-k\right]$$

A 1D wavelet transform was applied to each column, $A_{h}(:,j)$ and $D_{h}(:,j)$, resulting in four sub-bands, where the diagonal detail component (cD) is expressed as follows:

(16) $$cD\left(i,j\right)=\sum_{k}D_{h}\left(k,j\right)*h\left[2j-k\right]$$

Based on lung cancer images, we calculated the mean and variance of the wavelet transform cD component as texture parameters.

#### 2.4.3. Genetic Features

##### CNV Parameters

CNV affects gene expression by activating oncogenes or disabling tumor-suppressor genes, thereby promoting cancer development [37,38]. CNVs are crucial factors in tumorigenesis [39,40].

The original CNV dataset was merged into a seg file, and the corresponding marker file was downloaded for preprocessing. These two files were then processed using the GISTIC software (Version 3.9.11 prerelease; https://cloud.genepattern.org/gp/pages/index.jsf accessed on 19 March 2025) to obtain CNV data for different genes in all patients.

The values 0, 1, and 2 represent the amplitude threshold categories in CNVs that describe the extent of copy number changes. They are interpreted as follows:

0: t < 0.1 indicates little to no significant copy number changes at this location. This typically indicates that the gene copy number is close to normal.

1: 0.1 < t < 0.9 indicates a moderate degree of copy number change at this location. Changes within this range indicate a slight increase or decrease in the gene copy number.

2: t > 0.9 indicates a significant copy number change at this location. This often implies substantial copy number amplification or large-scale gene loss, which can greatly affect gene expression or function.

##### SNV Parameters

SNVs are mutations involving single-nucleotide changes in the normal human genome that lead to deletions, insertions, or substitutions. Tumorigenesis is closely associated with SNVs [41,42].

We extracted the following information for SNVs: TCGA Identity (TCGAID) (Tumor_Sample_Barcode), gene (Hugo_Symbol), and variant classification (Variant_Classification). The variant classification was processed, where mutation types “Missense”, “Nonsense”, “Nonstop”, “Translation_Start_Site”, “Frame_Shift_Del”, “Frame_Shift_Ins”, “In_Frame_Del”, “In_Frame_Ins”, and “Splice_Site” were considered as non-synonymous variants and assigned a value of 1. Mutation types “3′UTR”, “5′UTR”, “3′Flank”, “5′Flank”, “Silent”, “Intron”, “IGR”, “RNA”, and “Splice region” were considered synonymous variants and assigned a value of 0.

##### mRNA Parameters

mRNA directly or indirectly influences gene translation, reflecting the pathological state of tissues. Therefore, the detection of changes in intracellular mRNA levels can provide physiological evidence for early disease detection.

Based on the previous processing, we finally merged the clinical, nuclear, and genetic features of LUAD and LUSC based on the unique TCGAID to generate a comprehensive dataset.

### 2.5. Correlation Coefficient Analysis

The PCC (r) reflects the strength of the linear relationship between two variables, with values ranging from −1 to 1. An r value of 1 denotes a perfect positive correlation, 0 denotes no correlation, and −1 denotes a perfect negative correlation [43]. The formula for the PCC is below, where r represents the PCC; $x_{i}$ and $y_{i}$ are the ith data points of variables X and Y, respectively; $\bar{x}$ and $\bar{y}$ are the mean values of X and Y, respectively; and n is the number of data points:

(17) $$r=\frac{\sum_{i=1}^{n}\left(x_{i}-\bar{x}\right)\left(y_{i}-\bar{y}\right)}{\sqrt{\sum_{i=1}^{n}{\left(x_{i}-\bar{x}\right)}^{2}\sum_{i=1}^{n}{\left(y_{i}-\bar{y}\right)}^{2}}}$$

We used PCCs to analyze the relationships between all factors and survival times at 1, 2, and 3 years, as well as between factors and lung cancer subtypes. Based on the correlation coefficient values, factors with minimal impact were excluded. We retained features with an absolute correlation coefficient (|r|) greater than 0.05 and *p* < 0.05 to ensure statistical significance.

### 2.6. Machine- and Deep-Learning Models

In selecting machine- and deep-learning models, the following questions are considered: (1) What machine- or deep-learning models are suitable for classifying subtypes and predicting the overall survival of lung cancer? (2) Which model can achieve higher prediction accuracy? We selected the following models, which are useful for effectively reducing overfitting, can handle imbalanced datasets, support rapid iteration and optimization, and have high adaptability to tabular datasets: LightGBM, XGBoost, RF, AdaBoost, MLP, TabNet, and CNN. Based on the available nuclear, clinical, and genetic datasets, we assessed whether these models perform optimally in lung cancer subtype classification and survival prediction.

All the models were built using Anaconda 2023.9, Python 3.9.18, and PyTorch 1.8.0+cu111, with PyCharm 2022.3 as the integrated development environment. The hardware setup included an Intel(R) Xeon(R) W-2265 CPU (24 cores), Hynix HMA84GR7AFR4N-VK RAM (64 GB), and an NVIDIA GeForce RTX 3060 GPU (12 GB).

In addition, the Optuna library (Version: 3.6.1, https://optuna.readthedocs.io/zh-cn/stable/ accessed on 19 March 2025), an effective and widely used tool for parameter tuning of machine- or deep-learning models [44], was employed to select the optimal hyperparameters within the set range for predicting OS at 1, 2, and 3 years, as well as NSCLC subtypes, mainly LUAD and LUSC. The AUC, accuracy, recall, precision, F1-score, and learning duration of the different models were compared.

#### 2.6.1. Construction of the Input Dataset

The training (60%), validation (20%), and testing (20%) datasets were randomly divided based on different TCGAIDs. In other words, out of the total 1252 TCGAIDs, 752, 250, and 250 TCGAIDs were placed in the training, validation, and testing datasets, respectively. Differences in image staining characteristics can lead to variations in the number of nuclei segmented from each tile image, which can introduce bias in the analysis and imbalance in model training. To address this, we balanced the data by standardizing the sample size to an average of 200 nuclei (200 is the median number of nuclei for all TCGAIDs) per TCGAID dataset. We used two methods:

Random under-sampling: For TCGAIDs with more than 200 cell nuclei, we randomly selected 200 nuclei to avoid bias from excessive data.

Random over-sampling: For TCGAIDs with fewer than 200 cell nuclei, we randomly duplicated the existing nuclei to reach 200 nuclei, ensuring consistency in the sample size and avoiding instability in model training owing to insufficient samples, thus preserving the original data distribution characteristics. A total of 625 TCGAIDs with fewer than 200 nuclei underwent random over-sampling.

#### 2.6.2. Machine-Learning Models

LightGBM is an efficient distributed boosting algorithm with a gradient-boosting framework that can quickly process large-scale data [45]. It supports the optimal splitting strategy for categorical features, enabling direct processing of discrete variables (such as TNM stage) in clinical data without additional encoding. It also has a mechanism for handling the issue of missing values commonly seen in genetic data, avoiding information loss caused by data preprocessing. When dealing with complex and high-dimensional data such as images, clinical information, and genetic features, LightGBM leverages fast training and efficient feature selection to improve classification and prediction accuracy while maintaining computational efficiency. Table 2 lists the values or types used for each parameter during optimization. The model achieved the highest accuracy with the following parameters: num_leaves = 31, learning_rate = 0.08, num_iterations = 180, max_bin = 285, and boosting_type = “dart”.

XGBoost integrates regularization to prevent overfitting and facilitates parallel processing to enhance computational efficiency [46]. It supports custom loss functions and evaluation indicators and flexibly adapts to complex tasks such as lung cancer subtype classification and survival prediction. It is capable of fully exploring the nonlinear relationships between nuclear, clinical, and genetic features and quickly identifying important factors through its split-point optimization and tree-structure search strategy, generating prediction models with strong generalization ability, thus making it particularly suitable for handling complex classification and prediction tasks. The model achieved the highest accuracy with the following parameters: max_depth = 6, eta = 0.3, gamma = 0, min_child_weight = 3, colsample_bytree = 0.5, subsample = 0.5, lambda = 1, and alpha = 0.01 (Table 2).

RF is an ensemble learning method that improves model prediction performance by building multiple decision trees and voting on their results [47]. It does not require strict data distribution assumptions but naturally supports mixed-type input features (such as continuous clinical parameters and categorical TNM staging) and adaptively handles missing values through a majority voting mechanism, simplifying the data preprocessing process in multi-center studies. It can effectively reduce overfitting through random sampling and automatically identify and select key features, making it suitable for handling complex datasets and high-dimensional features. The model achieved the highest accuracy with the following parameters: n_estimators = 100, min_samples_split = 5, max_depth = 30, and min_samples_leaf = 2 (Table 2).

AdaBoost is a boosting algorithm that creates a strong classifier by incorporating multiple weak classifiers (typically decision trees) through weighted voting [48]. It dynamically adjusts the weights based on the error rate of each classifier, thereby gradually improving the classification performance of the model. AdaBoost excels in handling class imbalance in the data, particularly when sample sizes are uneven. In addition, despite its relatively high sensitivity to noise, AdaBoost can partially suppress the influence of measurement errors in clinical data and maintain the stability of subtype classification by limiting the complexity of weak classifiers and dynamically adjusting the sample weight distribution. The model achieved the highest accuracy with the following parameters: learning_rate = 0.1, algorithm = “SAMME.R”, and n_estimators = 200 (Table 2).

#### 2.6.3. Deep-Learning Models

MLP is a classical feedforward artificial neural network. It is trained using a backpropagation algorithm, which adjusts the weights of connections through gradient descent to minimize the error between the predicted and actual values [49]. Due to its flexibility, MLP can automatically adapt to various combinations of input features, making it effective in extracting latent predictive information, especially when handling complex genetic and image data. Table 3 lists the values or types used for each parameter during optimization. The model achieved the highest accuracy with the following parameters: max_iter = 200, hidden_layer_sizes = (100, 100), activation = “relu”, alpha = 0.1, solver = “adam”, and learning_rate_init = 0.01.

TabNet is a deep-learning model specifically developed for tabular data that combines the representation learning capabilities of neural networks with the interpretability of decision tree models [50]. It utilizes a sequential attention mechanism that dynamically selects the most relevant features at each decision step, allowing the model to focus on key variables. This selective learning feature enhances both predictive accuracy and interpretability, making TabNet particularly effective for structured data analysis. In this study, the factors were integrated into a tabular format. TabNet used an attention mechanism to prioritize key features, which allowed for the effective identification of important variables in the tabular dataset, thus improving both model accuracy and interpretability. The model achieved the highest accuracy with the following parameters: n_a = 16, n_d = 16, gamma = 1.3, n_steps = 3, n_shared = 4, n_independent = 2, lambda_sparse = 0.0001, and momentum = 0.1 (Table 3).

CNN refers to a one-dimensional CNN (1D-CNN). It is suitable for feature extraction from sequential and tabular data [51]. The 1D convolution kernel shares the weight when sliding on the sequence data, which significantly reduces the number of parameters. By using multiple layers of convolution and pooling operations, 1D-CNN can automatically learn and extract key features, helping to improve the accuracy of lung cancer subtype classification and survival prediction [52]. The model achieved the highest accuracy with the following parameters: out_channels1 = 65, out_channels2 = 153, and learning_rate = 0.00008 (Table 3).

#### 2.6.4. Evaluation of the Models

We used six evaluation metrics—accuracy, precision, recall, F1-score, AUC, and learning duration—to evaluate the performance of the models in predicting OS of patients at 1, 2, and 3 years and in classifying LUAD and LUSC. The formulas of the metrics are provided below, with *TP*, *FP*, *TN*, and *FN* denoting the number of true-positive, false-positive, true-negative, and false-negative samples, respectively.

Accuracy represents the proportion of correctly predicted samples out of all samples, providing an intuitive measure of the model’s overall effectiveness in multiclassification tasks.

(18) $$Accuracy=\frac{TP+TN}{TP+TN+FP+FN}$$

Precision is the ratio of samples predicted as belonging to a certain class that truly belongs to that class and measures the accuracy of positive predictions.

(19) $$Precision=\frac{TP}{TP+FP}$$

Recall is the ratio of samples that actually belong to a certain class to those that are accurately predicted as belonging to that class, quantifying the model’s capability in detecting positive instances from all positive examples.

(20) $$Recall=\frac{TP}{TP+FN}$$

F1-score is the harmonic mean of the precision and recall, offering a balanced assessment of the model’s effectiveness in classification tasks.

(21) $$F1-score=\frac{2*Precision*Recall}{Precision+Recall}$$

AUC is the area under the receiver operating characteristic (ROC) curve, which is a critical tool for evaluating the effectiveness of classification models. AUC values approaching 1 indicate superior model performance.

Finally, we used the model learning duration in the aforementioned hardware environment as another performance metric. This index reflects the computational efficiency and feasibility of the model for practical applications, particularly large-scale data processing.

## 3. Results

### 3.1. Segmentated Lung Cancer Cell Nuclei

#### 3.1.1. Tile Images for Annotation

**Figure 3:**
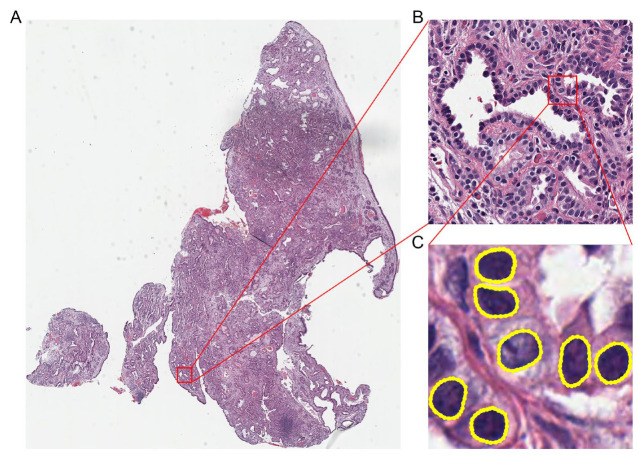
OpenSlide segments the entire lung tissue slide image into 1024 × 1024 tile images. (**A**) The entire lung tissue slide image (magnification: ×25), (**B**) one of the segmented tile images (magnification: ×400), and (**C**) the labeled nuclei circled out by yellow in LabelMe (magnification: ×400).

Lung tissue images downloaded from the TCGA were in the SVS format (Figure 3). We used OpenSlide to extract the level 0 image from the pyramid, which was the largest and clearest original image and segmented it into 1024 × 1024 tile images. The blank tiles with no valid data were excluded. Ultimately, 1067 original LUAD images were segmented into 617,452 tiles, and 1084 original LUSC images were segmented into 447,778 tiles. Using LabelMe software, we further annotated the positions and shapes of lung cancer cell nuclei in 3526 selected tiles with good staining quality, resulting in the corresponding mask images.

#### 3.1.2. Segmentation of Lung Cancer Cell Nuclei and Post-Processing

**Figure 4:**
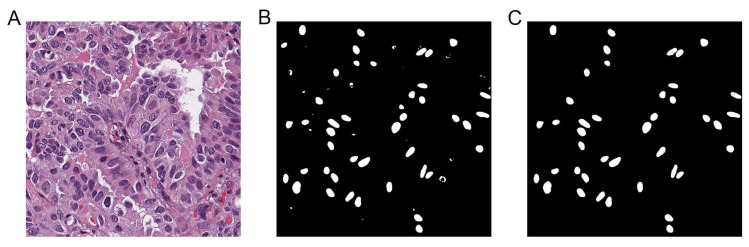
Automated segmentation of lung cancer cell nuclei based on UNet. (**A**) A lung cancer tile image, (**B**) the initial mask image predicted by the UNet model, and (**C**) the mask image after post-processing. Magnification: ×400.

As shown in Figure 4, the UNet model was employed to segment the cell nuclei in the lung cancer tile images using the training nuclei obtained from LabelMe (Figure 4A). After segmentation (Figure 4B), Otsu’s thresholding method was applied to remove nuclei with areas smaller than the area threshold and compactness greater than the compactness threshold. The resulting mask images of well-segmented nuclei were retained (Figure 4C).

### 3.2. Construction of a Multidimensional Dataset Incorporating Clinical, Nuclear, and Genetic Features

Using the unified TCGAID, the clinical, nuclear, and genetic features of LUAD and LUSC were merged into a comprehensive dataset. The specific factors included in this dataset are listed in Table 4.

After removing genetic factors with values of zero, we included 20 nuclear, 7 clinical, 15 CNV, 27 SNV, and 26 mRNA factors. For lung cancer subtype classification, we selected 46 factors from histopathological images and mRNA genetic analysis as independent variables, considering the complexity of feature acquisition and the convenience of practical application. mRNA is relatively easy to obtain and can provide gene expression data reflecting the biological characteristics of tumor tissues, thus supporting the model.

For survival prediction, we selected all 95 factors as independent variables for predicting OS at 1, 2, and 3 years. Table 5 presents the dependent variables.

### 3.3. Heatmap of Correlation Coefficients

**Figure 5:**
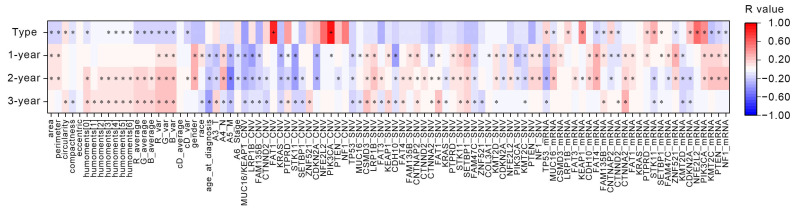
Heatmap of correlation coefficients. Features with |r| > 0.05 and *p* < 0.05 are marked with asterisks (*). High correlation features (|r| > 0.8) are marked with “+”.

Figure 5 presents the correlation coefficient heatmap of all original data before selection, where many values with low correlations and small absolute values can be observed. R-values above 0.05 are considered to indicate a positive/direct relation between the factors [43]; thus, after PCC analysis, features with |r| > 0.05 and *p* < 0.05 were retained and marked with asterisks (*) in the figure.

For lung cancer subtype classification, four nuclear features and eight mRNA features were removed, leaving 34 factors.

For 1-year survival prediction, 15 nuclear features and 19 genetic features were removed, leaving 61 factors. For a 2-year survival prediction, five nuclear features, one clinical feature, and 17 genetic features were removed, leaving 72 factors. For 3-year survival prediction, five nuclear features, two clinical features, and 33 genetic features were removed, leaving 55 factors.

### 3.4. Lung Cancer Subtype Classification and OS Prediction Based on Machine- and Deep-Learning Models

#### 3.4.1. Input Dataset for Modeling

We balanced the number of nuclei from each TCGA sample and selected highly correlated factors based on the PCCs to ensure balanced data distribution and provide effective features for model training. As shown in Table 6, the final dataset included 250,400 records, with 105,000 and 145,400 records from 525 and 727 patients with LUAD and LUSC, respectively. Among them, 150,400 records from 752 different TCGAIDs (315 patients with LUAD and 437 patients with LUSC) served as the training set, and two groups of 50,000 records from 250 different TCGAIDs (145 patients with LUAD and 105 patients with LUSC) were used as the validation and testing sets, respectively.

#### 3.4.2. Lung Cancer Subtype Classification

**Figure 6:**
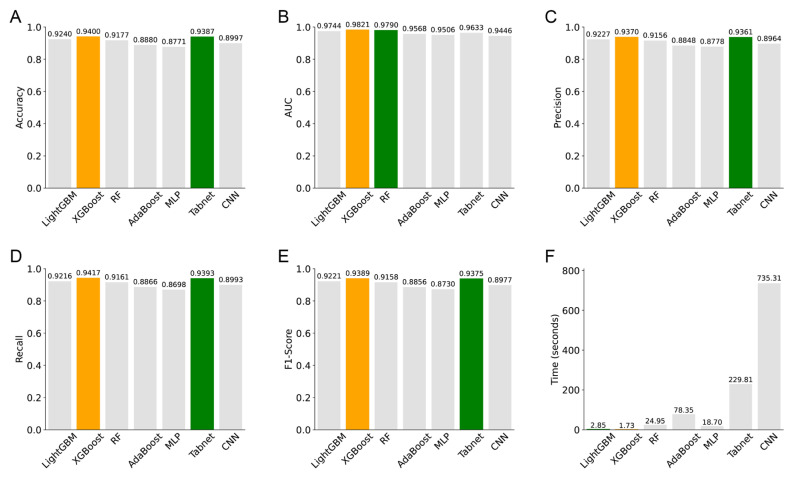
Performance evaluation of the seven models for lung cancer subtype classification. (**A**) accuracy, (**B**) AUC, (**C**) precision, (**D**) recall, (**E**) F1—score, (**F**) time in learning duration.

**Figure 7:**
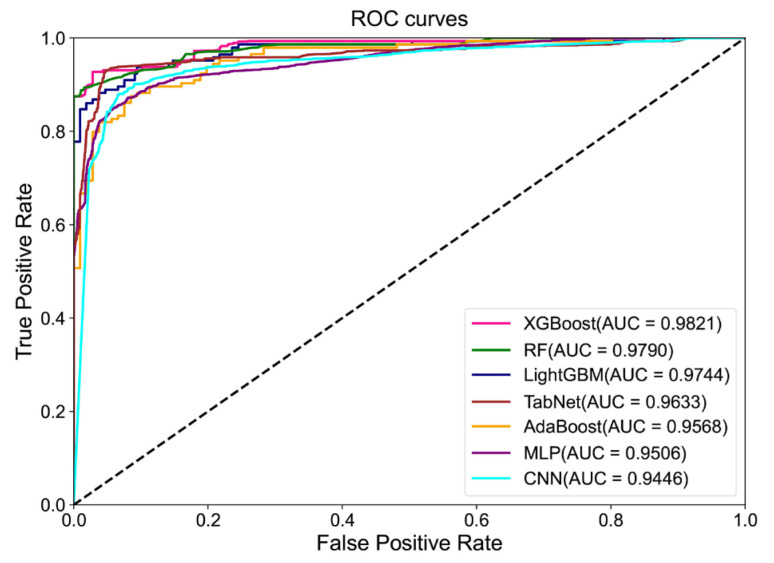
ROC curves of the seven models for lung cancer subtype classification.

Figure 6 shows the bar plots of the evaluation indices of the seven models (LightGBM, XGBoost, RF, AdaBoost, MLP, TabNet, and CNN) for lung cancer subtype classification. The indices included accuracy, precision, recall, F1-score, AUC, and learning duration. Yellow bars indicate the best performance for each index, and green bars indicate the second-best performance. Figure 7 shows the ROC curves of the seven models for lung cancer subtype classification. Figure S1 shows the confusion matrices of these seven models in classifying lung cancer subtypes.

For the classification of NSCLC subtypes, XGBoost performed the best across all six evaluation indices: accuracy, AUC, precision, recall, F1-score, and learning duration, with values of 0.9400, 0.9821, 0.9370, 0.9417, 0.9389, and 1.73 s, respectively. TabNet followed closely, although its learning duration was significantly longer. LightGBM and RF also exhibited good overall performance, ranking just behind TabNet. Finally, the CNN, MLP, and AdaBoost models demonstrated poorer performance in all accuracy-related metrics, with CNN’s learning duration being particularly long.

#### 3.4.3. Survival Prediction

**Figure 8:**
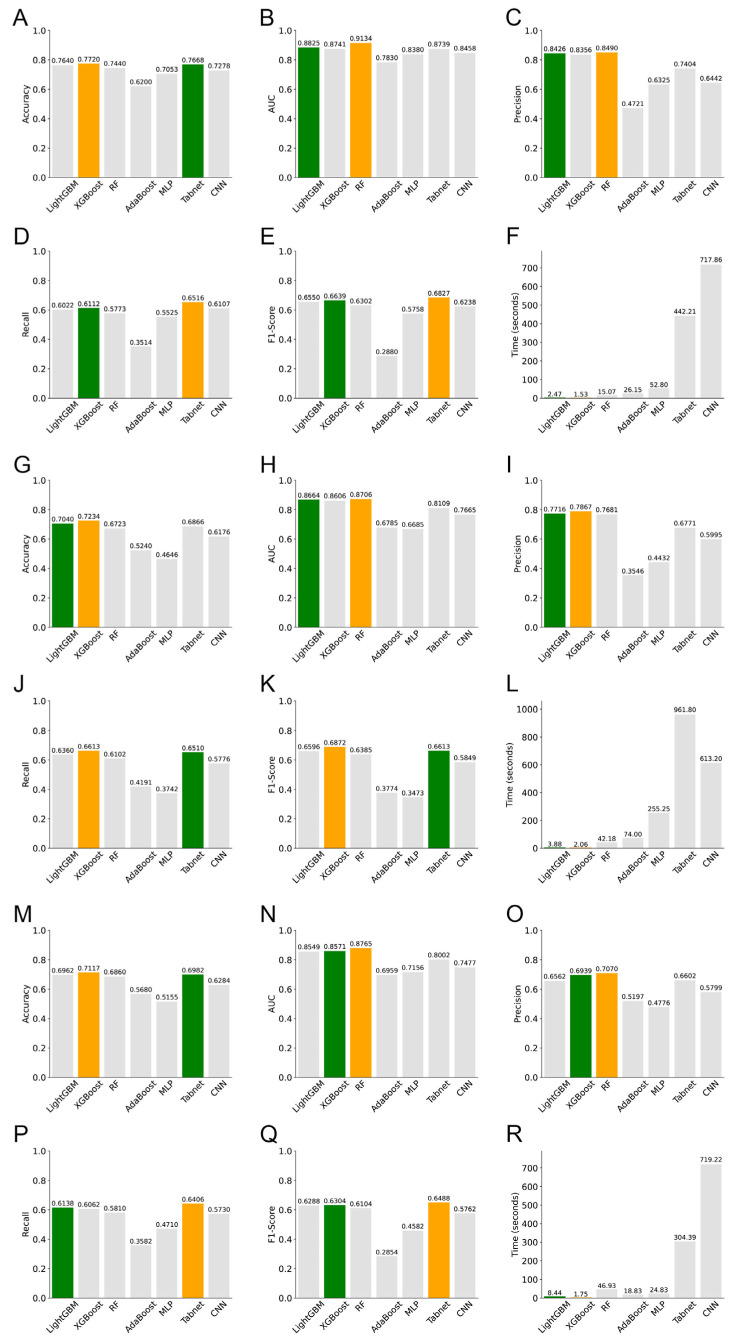
Performance evaluation of the seven models for (**A**–**F**) 1-, (**G**–**L**) 2-, and (**M**–**R**) 3-year OS predictions.

**Figure 9:**
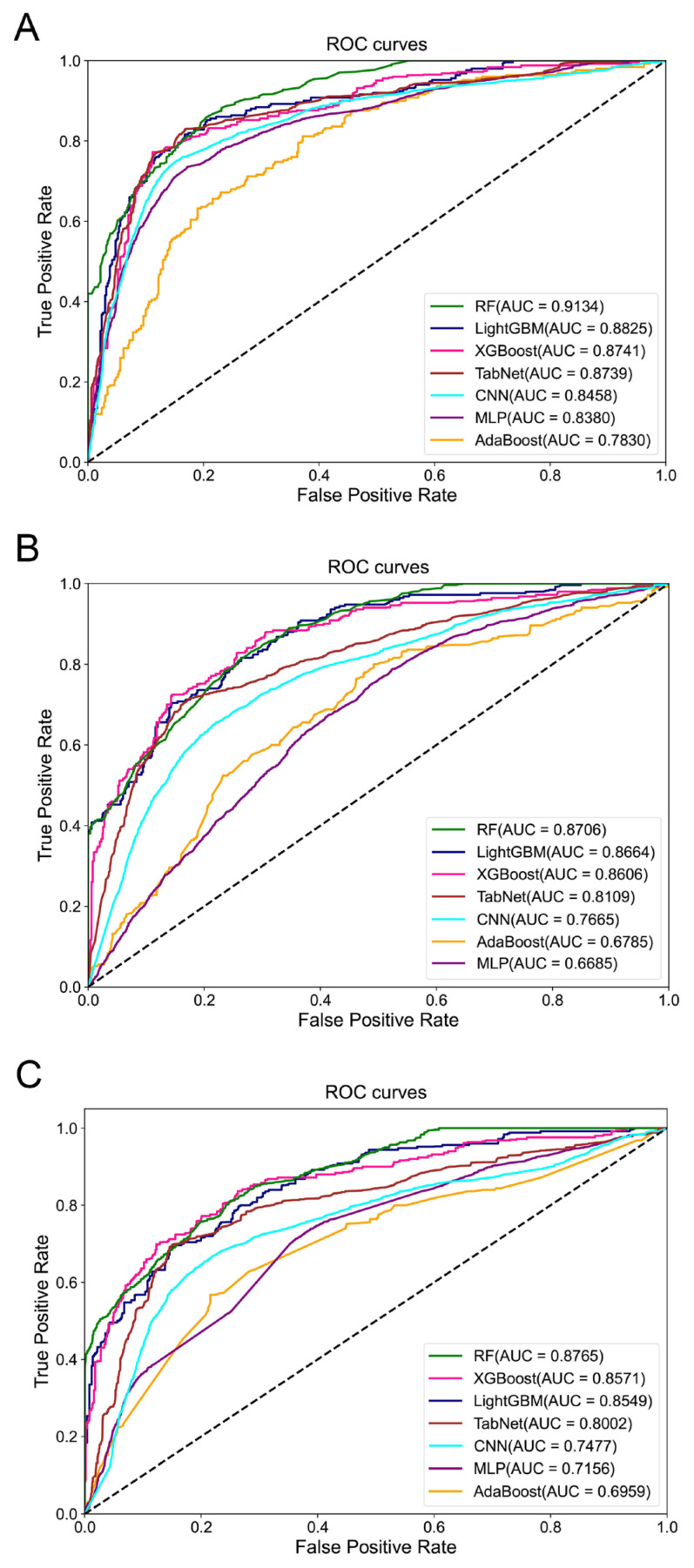
ROC curves of the seven models for (**A**) 1-, (**B**) 2-, and (**C**) 3-year survival predictions in patients with lung cancer.

Figure 8 and Figure 9 show bar plots of the evaluation indices and the ROC curves for survival predictions at 1, 2, and 3 years using the seven models, respectively. Yellow bars indicate the best performance for each index, and green bars indicate the second-best performance in Figure 8. Figure S2 shows the confusion matrices of these seven models.

In predicting survival rates at 1, 2, and 3 years, we observed that XGBoost achieved the highest accuracy, whereas RF excelled in terms of AUC. Both RF and XGBoost performed well in terms of precision. For recall and F1-score, TabNet and XGBoost stood out. XGBoost also exhibited the best performance in terms of learning duration. Notably, AdaBoost and MLP performed poorly in all aspects except the learning duration. TabNet and CNN exhibited excessive learning durations that were several hundred times longer than that of XGBoost, which was the fastest. Overall, XGBoost exhibited the best performance in OS predictions. For the 1-year prediction, the accuracy, AUC, precision, recall, F1-score, and learning duration of XGBoost were 0.7720, 0.8741, 0.8356, 0.6112, 0.6639, and 1.53 s, respectively; for the 2-year prediction, the corresponding values were 0.7234, 0.8606, 0.7867, 0.6613, 0.6872, and 2.06 s, respectively; for the 3-year prediction, the corresponding values were 0.7117, 0.8571, 0.6939, 0.6062, 0.6304, and 1.75 s, respectively.

## 4. Discussion

Our study employed seven models—four machine-learning models (LightGBM, XGBoost, RF, and AdaBoost) and three deep-learning models (MLP, TabNet, and CNN)—to conduct an in-depth analysis of survival prediction and subtype classification of patients with NSCLC, aiming to provide scientific evidence for the diagnosis and treatment of lung cancer. Our study was the first to incorporate the characteristics of nuclei and the genetic information of patients to predict the subtypes and OS of patients with lung cancer. The combination of different factors and the usage of AI methods increased the predictive accuracy compared to previous studies [13,14,15,16,17,18,19,22,23]. Determining the subtypes of lung cancer in patients is important for selecting treatment options; meanwhile, more attention can be paid to patients who are screened as high-risk. Some subtype classification and survival prediction studies showed limitations related to insufficient sample sizes and compromised model accuracy [13,14,15,16,17,18,19,22,23]. Some studies also relied only on data from a single institution, which further restricted the applicability of the models used [8,9,13,15,16,17]. The characteristics of images acquired at different medical centers can vary widely, and consequently, the generalization ability of a prediction model trained using data from only one center tends to be weak [53]. Our research utilized the TCGA database, which contains data from multiple cancer research institutions, and we obtained data from 1252 patients with NSCLC (including 525 patients with LUAD and 727 patients with LUSC). The training, validation, and testing datasets were generated by dividing the TCGAIDs in a 60%:20%:20% ratio to ensure the independence of each dataset.

The majority of previous studies focusing on subtype classification or survival prediction were based on PET/CT images. For example, Han et al. used VGG16 for classification based on the first-order intensity statistics and texture features of PET/CT images from Peking University Cancer Hospital, achieving an AUC of 0.903 [8]. Bicakci et al. collected the PET/CT images of 94 lung cancer cases (including 38 LUAD and 56 LUSC cases) from Acıbadem Kayseri Hospital in Turkey and used the VGG19 model for classification, achieving an AUC of 0.69 [13]. Marentakis et al. downloaded the preprocessed CT images of 102 lung cancer cases (including 48 LUAD and 54 LUSC cases) from the TCIA platform. They used a long short-term memory + inception model based on four groups of different CT radiomic features (statistical features of the first order, shape, texture features, and wavelet features) for lung cancer subtype classification, achieving an AUC of 0.78 and an accuracy of 74% [14]. Bashir et al. collected the data from 64 LUAD and 42 LUSC cases from a local hospital in the UK and used an RF model to predict the subtypes on the basis of CT radiomics, nodule semantics, and background parenchymal features, achieving an AUC of 0.82 [15]. Overall, the best AUC value for subtype classification was around 0.9. For OS prediction, Jha et al. collected CT imaging data of 200 NSCLC cases from the Tata Memorial Hospital in Mumbai, India. Based on radiomic features, they used an RF model to predict the 2-year survival rate, achieving an accuracy of 81% [22]. Regarding image analysis, many previous studies manually delineated entire tumor regions in images [14,16,19,21,22] or used sliced H&E images [11,12]. Stained tissues in histological images not only contain nuclei but also various other structures, such as connective tissue or blood vessels. Using entire images may neglect detailed information or lead to distraction by these structures. There are limited studies using features of nuclei to classify the lung cancer subtypes or predict the OS. In a previous study, the long-term versus short-term survival among patients with early-stage NSCLC was classified based on the spatial proximity and attributes (e.g., shape, size, and texture) of individual nuclei, yielding a mean AUC of 0.68 in the training cohort [29]. Alsubaie et al. characterized the morphometric features of tumor nuclei and found that they have a significant correlation with OS in LUAD. However, they did not propose a predictive model [54]. Because the cell nucleus can provide key information for identifying the presence or the stage of disease [55], we chose to focus on the nuclear features of lung cancer cells.

We downloaded SVS format images from the TCGA database and used OpenSlide to divide them into 1024 × 1024 pixel tiles. The UNet model can accurately and efficiently segment cell nuclei into new tile images by combining the location and shape information of lung cancer cell nuclei marked with LabelMe. Subsequent post-processing based on Otsu’s thresholding method was performed to eliminate fragmented parts and retain high-quality cell nucleus mask images. This automated segmentation approach improved processing efficiency and provided precise cell nucleus data for subsequent subtype classification and survival prediction. Following that, a total of 20 nuclear features were extracted from the histological images: R_average, G_average, B_average, R_var, G_var, B_var, area, perimeter, circularity, compactness, eccentric, Hu[0], Hu[1], Hu[2], Hu[3], Hu[4], Hu[5], Hu[6], cD_average, and cD_var. PCC analysis was conducted to filter and optimize the feature–factor system. After conducting the PCC analysis, a total of 16 nuclear variables—namely, area, perimeter, circularity, compactness, Hu[0], Hu[3], Hu[4], Hu[5], Hu[6], R_average, G_average, B_average, R_var, G_var, B_var, and cD_var—were found to be significantly correlated with subtypes of lung cancer, which implies that nuclear features could be used as indicators for subtype classification and OS prediction. Almost all the nuclear features and the mRNA levels of the mutated genes are important predictors for subtype prediction, indicating that there are differences in the morphological characteristics of cancer cell nuclei and the mRNA levels between the LUAD and LUSC samples. In addition, machine- and deep-learning models can reduce inconsistencies arising from pathologists’ subjective judgments, thus enhancing the reliability of diagnosis and assessment and reducing time and labor costs. By combining nuclear and mRNA features, we found that the XGBoost model was the best for subtype classification, achieving an accuracy of 94% and an AUC of 0.9821, which was a small breakthrough compared to previous studies.

OS is relatively complicated to predict as it can be affected by various factors, such as the stage at which the patient was detected, treatment options, and the patient’s mental status and responses to treatment. Some studies were based on PET/CT images [22], some were based on mutational genes [56], while others were merely based on the clinical characteristics of patients [24]. For example, a study using the Surveillance, Epidemiology, and End Results (SEER) database only focused on age, sex, race, TNM stage, and the number of positive lymph nodes without incorporating genetic characteristics. The predictive AUC was only 0.744 [24]. While some studies incorporated the genetic information of patients, the AUC values obtained were compromised. For example, Zhang et al.’s study [25] achieved an AUC value of 0.67 only. Other studies did not provide an AUC value [56]. In our study, we integrated clinical, nuclear, and genetic features, aiming to provide detailed structural and morphological information about the cancerous tissues, thus supporting the OS prediction to a large extend. After conducting the PCC analysis, a total of five nuclear variables—namely, area, perimeter, R_var, G_var, and B_var—were found to be significantly correlated with one-year survival of patients with lung cancer; a total of 15 nuclear variables—namely, area, perimeter, Hu[0], Hu[2], Hu[3], Hu[4], Hu[5], Hu[6], R_average, G_average, B_average, R_var, G_var, B_var, and cD_var—were found to be significantly correlated with two-year survival of patients with lung cancer; a total of 15 nuclear variables—namely, circularity, compactness, Hu[0], Hu[1], Hu[2], Hu[3], Hu[4], Hu[5], Hu[6], R_average, G_average, B_average, R_var, G_var, and B_var—were found to be significantly correlated with three-year survival of patients with lung cancer. In general, not only nuclear features but also clinical features and the SNV, CNV, and mRNA levels of the top 20 mutated genes are clinically important determinants for OS prediction. Regarding the methods for OS prediction, traditional linear methods such as nomograms can only address linear relationships, whereas machine- and deep-learning models can model nonlinear risk functions and capture complex features within high-dimensional datasets. We found that XGBoost also achieved the best overall performance for OS prediction regarding all the metrics: accuracy, AUC, precision, recall, F1-score, and learning duration. However, if we only took the AUC values into consideration, RF performed the best: its AUC values for predicting OS at 1, 2, and 3 years were 0.9134, 0.8706, and 0.8765, respectively, which were all above 0.87 (Figure 9). These results demonstrated that it was optimal to incorporate nuclear characteristics along with the genetic information of patients. Our study provides a more accurate and comprehensive model for distinguishing NSCLC subtypes and predicting OS based on the morphometric features of tumor nuclei.

Our results show that machine-learning has significant advantages over deep-learning when dealing with structured data. First, machine-learning algorithms can effectively process structured tabular data. Our dataset includes finely extracted features such as clinical, image, and genetic features. XGBoost, which is based on gradient-boosting trees, is particularly suitable for such structured data. In contrast, deep-learning models often rely more on automatically extracted features from raw data. Moreover, when handling tabular data, XGBoost typically outperforms deep-learning models such as TabNet [57]. Second, machine-learning performs better on small to medium-sized datasets. Due to the correlation among nuclei from the same patient, the effective sample size is 1252 rather than the total 250,400 data records. Although deep-learning has outstanding performance on large-scale data [58], at the patient level, our dataset is relatively small, which may not be sufficient to support complex deep-learning models. In comparison, machine-learning models exhibit stronger generalization ability on small to medium-sized datasets. We can further optimize XGBoost and RF models by tuning hyperparameters and employing parallel computing techniques to improve training efficiency.

Although our study showed improved AUC values for subtype classification and OS prediction in NSCLC cases, it still had some limitations. First, our dataset only utilized the TCGA database. Future studies could validate our model using other databases. However, the current open-source datasets, such as TCIA and SEER databases, do not provide all the genomic, imaging, and clinical data of patients. Future studies could collaborate with local hospitals to collect complete datasets and further validate our model. Second, for feature selection, we only used histopathological images, clinical information, and genetic features. Future studies could incorporate other features, such as metabolomics, proteomics, and immunomics, which may further enhance the predictive capabilities of the model. Third, in terms of correlation analysis, in addition to PCC, Spearman’s rank correlation analysis and RF feature importance assessment can be further introduced to comprehensively evaluate the importance of factors, enabling the accurate removal of factors with very low correlations. Fourth, in addition to employing multiple machines and deep-learning models, with advancements in algorithms and computational power, future studies could introduce self-supervised learning [59] to leverage unlabeled histopathological and genomic data for feature pretraining; reinforcement learning [60] to capture long-term dynamic associations by designing a cumulative reward function for survival prediction; transformers [61] to enhance the extraction of global contextual features from imaging and sequencing data; and graph neural networks [62] to model complex interactions between genetic, clinical, and imaging variables. Additionally, using an optimized UNet++ model instead of the basic UNet model may further enhance segmentation accuracy and robustness. In the future, a toolkit can also be developed based on Python to establish a fully automated workflow for the entire process, from inputting the sliced images and the clinical and genetic features of patients to outputting the results of the subtype and OS prediction. Overall, future research should emphasize the integration of multidimensional data and explore more suitable machine- and deep-learning models, which may further improve the accuracy of OS prediction and subtype classification in patients with NSCLC and provide more reliable support for clinical decision-making.

## 5. Conclusions

In this study, we used machine- and deep-learning methods to present models for subtype classification and OS prediction in patients with LUAD and LUSC based on histopathological nuclear images, clinical information, and genetic features. The results showed that XGBoost and RF performed the best in classifying subtypes and predicting OS in terms of AUC values, respectively, thus demonstrating the potential of using automated detection to reduce pathologist workloads and provide information for treatment. With the introduction of additional feature factors and the application of more advanced models, we anticipate further improvements in this field.

## Figures and Tables

**Table 1:** Mapping of numerical values to clinical characteristics.

Clinical Characteristics	0	1	2	3	4	5
Sex	/	Male	Female	/	/	/
Race	Unreported	American Indian or Alaska native	Asian	Black or African American	White	/
T	/	T1	T2	T3	T4	TX
N	/	N0	N1	N2	N3	NX
M	/	M0	M1	/	/	MX
Stage	/	Stage I	Stage II	Stage III	Stage IV	/
1_year_survial	Unknown	<1-year	>1-year	/	/	/
2_year_survial	Unknown	<2-year	>2-year	/	/	/
3_year_survial	Unknown	<3-year	>3-year	/	/	/

**Table 2:** Optimal hyperparameters for the machine-learning models.

Models	Parameters	Values/Types
LightGBM	num_leaves	**31**, 64, 128, 256
	learning_rate	0.01, 0.02, 0.04, **0.08**, 0.1
	num_iterations	100, 120, 140, 160, **180**, 200
	max_bin	255, 265, 275, **285**, 300
	boosting_type	“gbdt”, “**dart**”, “goss”
XGBoost	eta	0.01, 0.05, 0.1, **0.3**
	max_depth	3, **6**, 10
	min_child_weight	1, **3**, 5, 10
	gamma	**0**, 0.1, 0.5, 1
	subsample	**0.5**, 0.7, 1
	colsample_bytree	**0.5**, 0.7, 1
	lambda	0, 0.1, 0.5, **1**
	alpha	0, **0.01**, 0.1, 1
RF	n_estimators	**100**, 150, 200
	max_depth	5, 10, 20, **30**, 50
	min_samples_split	2, **5**, 10, 20
	min_samples_leaf	1, **2**, 5, 10
AdaBoost	n_estimators	50, 75, 100, 125, 150, 175, **200**
	algorithm	“**SAMME.R**”, “SAMME”
	learning_rate	0.01, 0.025, 0.05, 0.075, **0.1**

Note: Bold text represents the final selected parameters.

**Table 3:** Optimal hyperparameters for the deep-learning models.

Models	Parameters	Values/Types
MLP	hidden_layer_sizes	(50, 50), **(100, 100)**
	max_iter	100, **200**, 300
	activation	“**relu**”, “tanh”, “logistic”
	solver	“**adam**”, “sgd”, “lbfgs”
	alpha	0.0001, 0.001, 0.01, **0.1**
	learning_rate_init	**0.01**, 0.05, 0.1
TabNet	n_d	8, **16**, 32, 64
	n_a	8, **16**, 32, 64
	n_steps	**3**, 5, 7, 10
	gamma	**1.3**, 1.5, 1.7, 2.0
	n_independent	**2**, 3, 4, 5
	n_shared	2, 3, **4**, 5
	lambda_sparse	**0.0001**, 0.0005, 0.001
	momentum	0.02, **0.1**, 0.2, 0.3
CNN	out_channels1	[64, 256]: **65**
	out_channels2	[64, 256]: **153**
	learning_rate	[0.00001, 0.0001]: **0.00008**

Note: Bold text represents the final selected parameters.

**Table 4:** Multidimensional clinical, nuclear, and genetic factors.

Categories	Subcategories	Factors
Clinical	Basic information	Gender, Race, Age_at_diagnosis
	Tumor stage	T, N, M, Stage
Nuclear	Color	R_average, G_average, B_average, R_var, G_var, B_var
	Morphology	Area, Perimeter, Circularity, Compactness, Eccentric, Hu[0], Hu[1], Hu[2], Hu[3], Hu[4], Hu[5], Hu[6]
	Texture	cD_average, cD_var
Genetic	CNV	*TP53, MUC16, CSMD3, LRP1B, FAT3, KEAP1, CDH10, FAT4, FAM135B, CNTNAP2, CTNND2, CTNNA2, FAT1, KRAS, PTPRD, STK11, SETBP1, FAM47C, ZNF521, COL3A1, KMT2D, CDKN2A, NFE2L2, PIK3CA, KMT2C, PTEN, NF1*
	SNV	
	mRNA	

**Table 5:** Dependent variables for lung cancer subtype classification and OS prediction.

Dependent Variables	Descriptions	Categories
Type	Lung cancer subtypes	“LUAD” as 1, “LUSC” as 2
1-year	1-year survival	“Unknown” as 0, “<1-year” as 1, “>1-year” as 2
2-year	2-year survival	“Unknown” as 0, “<2-year” as 1, “>2-year” as 2
3-year	3-year survival	“Unknown” as 0, “<3-year” as 1, “>3-year” as 2

**Table 6:** Distribution of the training, validation, and testing datasets.

Types of Datasets	Total Number of Records	Number of Records from Cases of LUAD	Number of Records from Cases of LUSC	Proportions of Records
Training set	150,400	63,000	87,400	60%
Validation set	50,000	21,000	29,000	20%
Testing set	50,000	21,000	29,000	20%
Total	250,400	105,000	145,400	100%

## Data Availability

The data presented in this study are available at NIH GDC Data Portal (https://portal.gdc.cancer.gov/projects/TCGA-LUAD (accessed on 10 October 2022). and https://portal.gdc.cancer.gov/projects/TCGA-LUSC (accessed on 10 October 2022)).

## Supplementary Materials

The following supporting information can be downloaded at https://www.mdpi.com/article/10.3390/diagnostics15070872/s1. Figure S1: The confusion matrices of the seven models in classifying lung cancer subtypes; Figure S2: The confusion matrices of the seven models in predicting the OS of patients with LUAD and LUSC.

## Abbreviations

The following abbreviations are used in this manuscript:

NSCLC	non-small cell lung cancer
LUAD	lung adenocarcinoma
LUSC	lung squamous cell carcinoma
CT	computed tomography
PET	positron emission tomography
WSIs	whole-slide images
AUC	area under the curve
RF	random Forest
TCIA	the cancer imaging archive
TNM	tumor node metastasis
CNN	convolutional neural network
XGBoost	extreme gradient boosting
H&E	hematoxylin and eosin
AdaBoost	adaptive boosting
OS	overall survival
SEER	surveillance, epidemiology, and end results
TCGA	the cancer genome atlas
CNV	copy number variation
SNV	single nucleotide variant
CSS	current survival status
mRNA	messenger RNA
PCC	Pearson’s correlation coefficient
LightGBM	light gradient boosting machine
MLP	multilayer perceptron
TabNet	tabular neural network
TCGAID	TCGA identity
ROC	receiver operating characteristic
