# Supplementary material for: Integration of Nuclear, Clinical, and Genetic Features for Lung Cancer Subtype Classification and Survival Prediction Based on Machine- and Deep-Learning Models
Source: Diagnostics (Basel). 2025 Mar 28;15(7):872. doi: 10.3390/diagnostics15070872 (PMC11988547; doi:10.3390/diagnostics15070872)
Supplement: Supplementary file 1 [file diagnostics-15-00872-s001.zip › diagnostics-3514165-supplementary.pdf]

Supplementary Materials

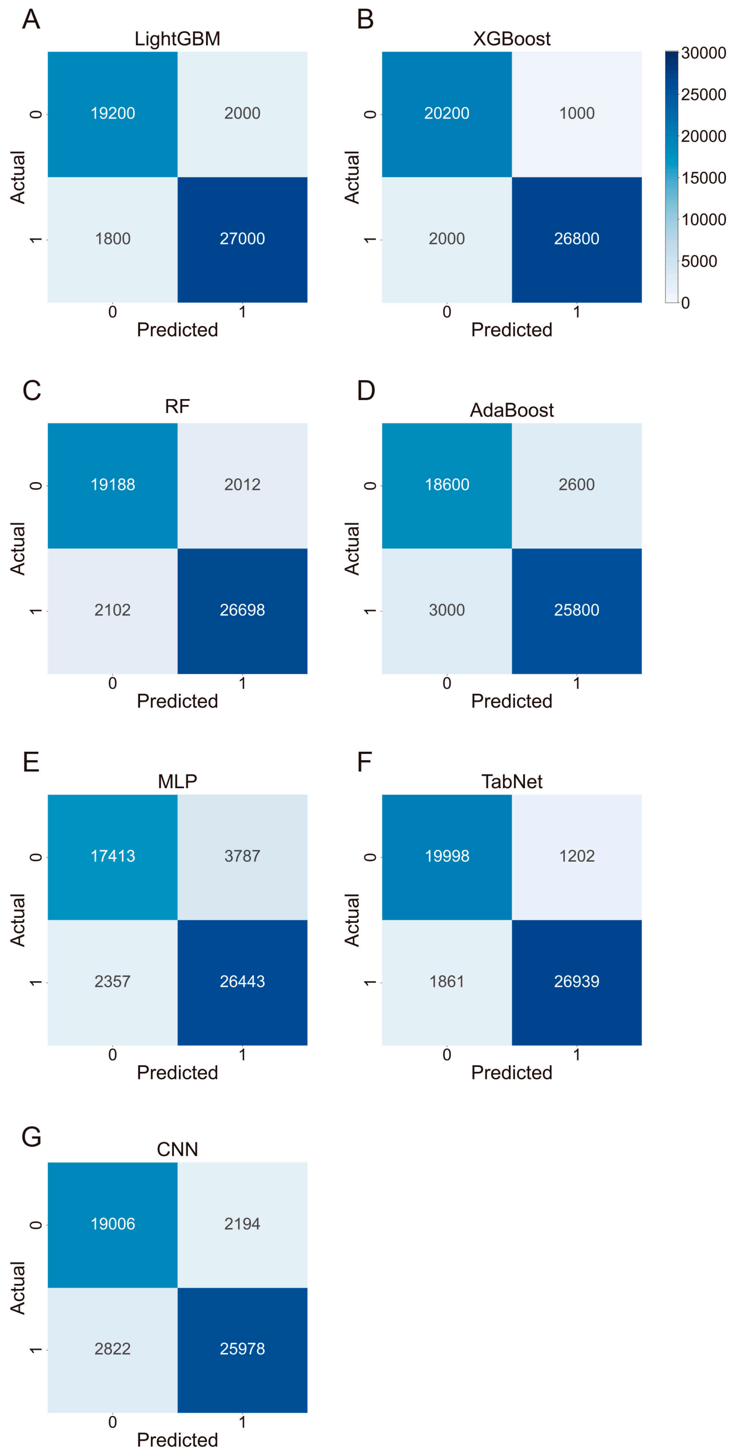

Figure S1. The confusion matrices of the seven models in classifying lung cancer subtypes.

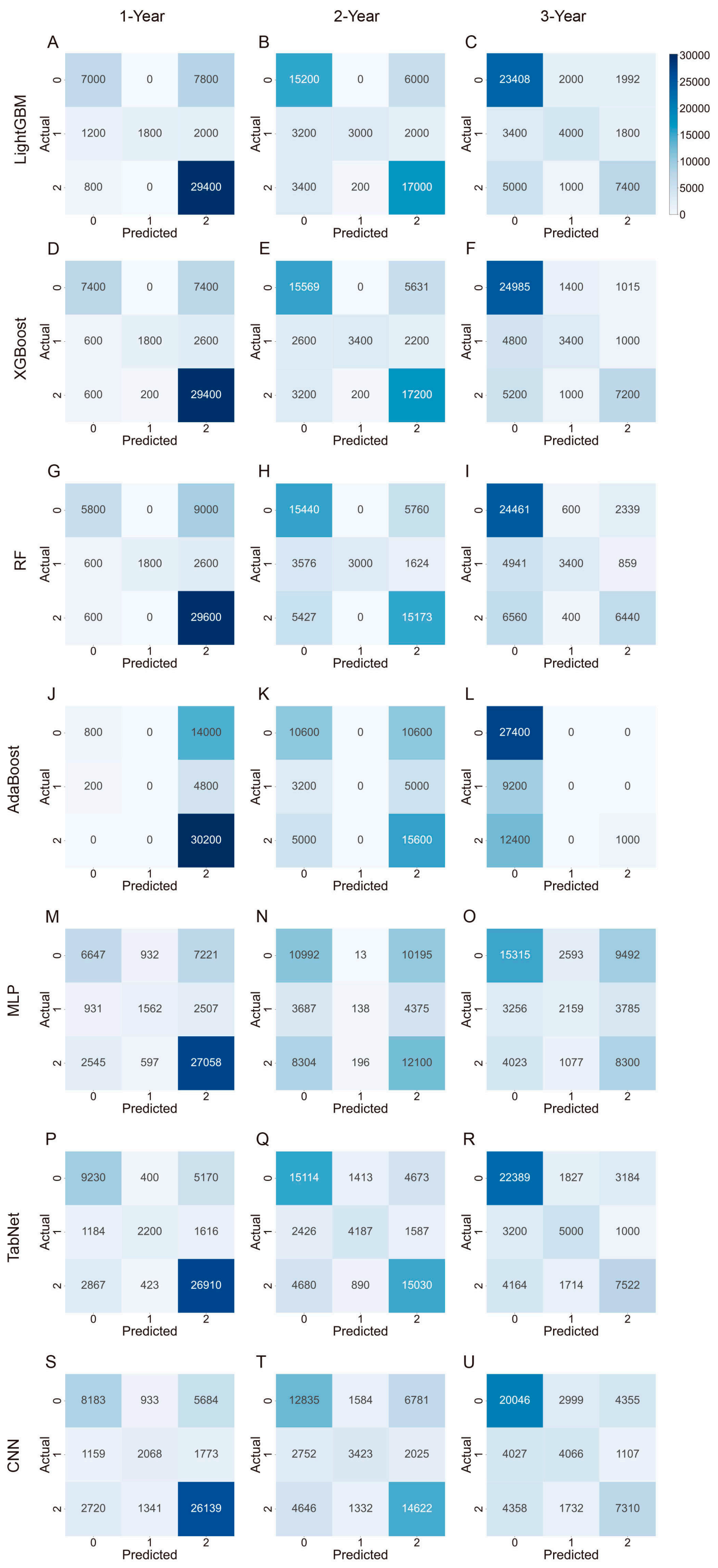

Figure S2. The confusion matrices of the seven models in predicting the OS of patients with LUAD and LUSC.
